# Association between weekend warriors and MASLD—a cross-sectional study of the NHANES database 2017–2020

**DOI:** 10.3389/fmed.2025.1531437

**Published:** 2025-04-02

**Authors:** Bo Yang Su, Song Wang, Tie Jun Liu, Yan Leng, Zhi Yuan Liu, Lu Liu, Zhuang Xiong

**Affiliations:** ^1^Changchun University of Chinese Medicine, Changchun, China; ^2^The Affiliated Hospital of Changchun University of Traditional Chinese Medicine, Changchun, China

**Keywords:** metabolic dysfunction-associated steatotic liver disease, weekend warrior, physical activity patterns, the National Health and Nutrition Examination Survey, cross-sectional study

## Abstract

**Background:**

The prevalence of metabolic dysfunction-associated steatotic liver disease (MASLD) continues to rise each year, posing a significant threat to people in their physical and mental health, as well as imposing a considerable economic burden on healthcare systems. Furthermore, physical activity (PA) is recognized as one of the effective strategies for the prevention of MASLD. However, the epidemiological evidence on the association between weekend warriors’ (WWs) exercise modes and MASLD is inconsistent. The primary objective of this study was to further investigate the association between weekend warriors and the prevalence of MASLD using the NHANES database.

**Methods:**

This study included a total of 4,671 participants from the National Health and Nutrition Examination Survey (NHANES) database. PA questionnaires were used to assess participants’ PA patterns, while vibration-controlled transient elastography (VECT) was used to assess the degree of hepatic steatosis, and other data were used to diagnose MASLD. Three distinct models were developed to compare the associations between various exercise patterns and the prevalence of MASLD through logistic regression, and to compare the differences between RA and WWs in the prevalence of MASLD.

**Results:**

There is a clear link between the involvement of WWs or RA participants and the lower prevalence of MASLD. In the final adjusted model, participants with a weekend warrior physical activity pattern (odds ratio [OR]: 0.511, 95% confidence interval [CI]: 0.373–0.701, *p* = 0.00.6) and those in the regular activity population (OR: 0.621, 95% CI: 0.512–0.754, *p*: 0.00.3) showed significantly lower risk ratios compared to individuals in the inactive and under-exercised populations, and this was statistically significant. Using the regular activity population as a reference, the risk of prevalence of MASLD in the weekend warrior group (OR: 0.857, 95% CI: 0.548–1.339, *p*: 0.516) indicates that no statistically meaningful disparity was observed between the two groups.

**Conclusion:**

In summary, our results demonstrate a significant correlation between WWs’ activity patterns and their risk of MASLD, and they indicate that these patterns can improve MASLD with benefits comparable to those of RA. This provides additional options for individuals with MASLD who are unable to meet the recommended criteria in the exercise guidelines, along with treatment options for clinicians.

## 1 Introduction

Currently, the leading cause of chronic liver disease worldwide is now non-alcoholic fatty liver disease (NAFLD) (1). Over the past decade—with further research advances in the complex physiological and pathological mechanisms of NAFLD—the fact that NAFLD is a multisystemic illness has increasingly come to light (2), meanwhile, the pathogenicity of NAFLD and its associated liver illnesses (liver cirrhosis, liver failure, and hepatocellular cancer) is significantly influenced by insulin resistance and metabolic dysfunction (3). In 2023, three notable international liver societies agreed to replace NAFLD with the nomenclature of metabolic dysfunction-associated steatotic liver disease (MASLD). The consistency between the two definitions is highly appropriate. According to relevant research, 99% of NAFLD patients meet the diagnostic criteria for MASLD (4). The nomenclature of MASLD not only better illustrates the physiological and pathological characteristics of this complex liver disease but also reflects the impact of cardiometabolic indicators, thereby increasing public understanding of the disease.

The development of MASLD is driven by various metabolic risk factors, including obesity, insulin resistance, hypertension, hyperlipidemia, and hyperglycemia (5).

MASLD is closely associated with both morbidity and mortality resulting from MASLD and its associated cardiovascular disorders, and it is recognized as a risk factor for adverse cardiovascular events. In addition, substantial epidemiological evidence indicates that MASLD is a risk factor for numerous extrahepatic cancers (such as malignancies of the breast, pancreas, esophagus, uterus, bladder, and colorectal regions, among others) (6), alongside hepatocellular carcinoma. Globally, the prevalence of MASLD has risen over the last three decades, and in 2019, nearly 30% of adults worldwide were expected to have it (7). With the rising prevalence of obesity, type-2 diabetes mellitus, and metabolic syndrome, MASLD has become more and more prevalent in the global population. A substantial and steadily rising global health and economic burden is posed by MASLD (8).

Currently, no specific medications have been approved to treat MASLD, and the principle of “prevention is better than cure” is particularly relevant for individuals with MASLD. Therefore, lifestyle changes remain an important aspect of combatting MASLD. The two main pillars of treatment for MASLD are dietary management and physical activity (8). First, dietary programs specifically involve a controlling diet to reduce calorie intake and eliminate ingredients that promote MASLD (i.e., processed foods and foods that contain large amounts of added fructose) (9). Second, physical activity is the best method to reduce an adult’s risk of MASLD. According to the World Health Organization (WHO) 2020 guidelines on physical activity and sedentary behavior (10), adults should engage in at least 150–300 min of moderate-intensity physical activity, 75–150 min of vigorous-intensity physical activity, or an equivalent combination of both, per week. Undoubtedly, these recommendations are effective in combatting MASLD, but in a prospective study of MASLD treatment through lifestyle changes, only 30% of the participants achieved weight loss through lifestyle changes by the end of the experiment over a 52-week period, and the reduction was only at the level of 5% of the original body weight, which was far from the requirement of a 10% reduction in body weight (11). This essentially represents the dilemma faced by some MASLD patients who struggle to achieve their weight-loss goals. Additionally, the available research indicates that approximately 80% of American adults and adolescents do not meet the minimal recommended weekly PA level (12). With the accelerated pace of global society, adherence to regular activity becomes increasingly challenging. Recently, a new model of physical activity has emerged: the “weekend warriors (WWs),” which encourages engaging in moderate-intensity exercise for at least 150 min only 1–2 times per week, as it better accommodates busy lifestyles. Numerous studies have also demonstrated that the WWs model achieves similar benefits to RA in reducing all-cause and cause-specific mortality (13), decreasing the risk of cancer death (14), lipid accumulation products (LAP) (15), and the cardiometabolic index (CMI) (16), as well as improving abdominal and general obesity (17) and frailty indices in adults (18). It also contributes to a decrease in diabetes mellitus (19) and risk of depressive episodes (20).

Our primary goal in this study has been to use the NHANES dataset to explore the association between various PA patterns and MASLD. Our findings might serve as a PA reference for MASLD patients seeking for an appropriate PA pattern, and to further confirm whether there is a relevance between exercise patterns in WWs or RA and the low prevalence of MASLD.

## 2 Methods

### 2.1 Data source

This cross-sectional study used the NHANES database operated by the Centers for Disease Control and Prevention (CDC), which assess the health and nutritional status of the US population (21). Data collection methods included face-to-face or telephone interviews, detailed questionnaires, laboratory analyses, and physical examinations. The NHANES protocol received approval from the National Ethical Review Board for Health Statistics, and written informed consent was obtained from all participants. All NHANES data were de-identified and the survey data and methods can be accessed on the NHANES official website (accessed till 28 October 2024). Since this study used publicly available data in compliance with the Declaration of Helsinki, ethics committee approval was not necessary.

### 2.2 Study cohort

**Figure 1:**
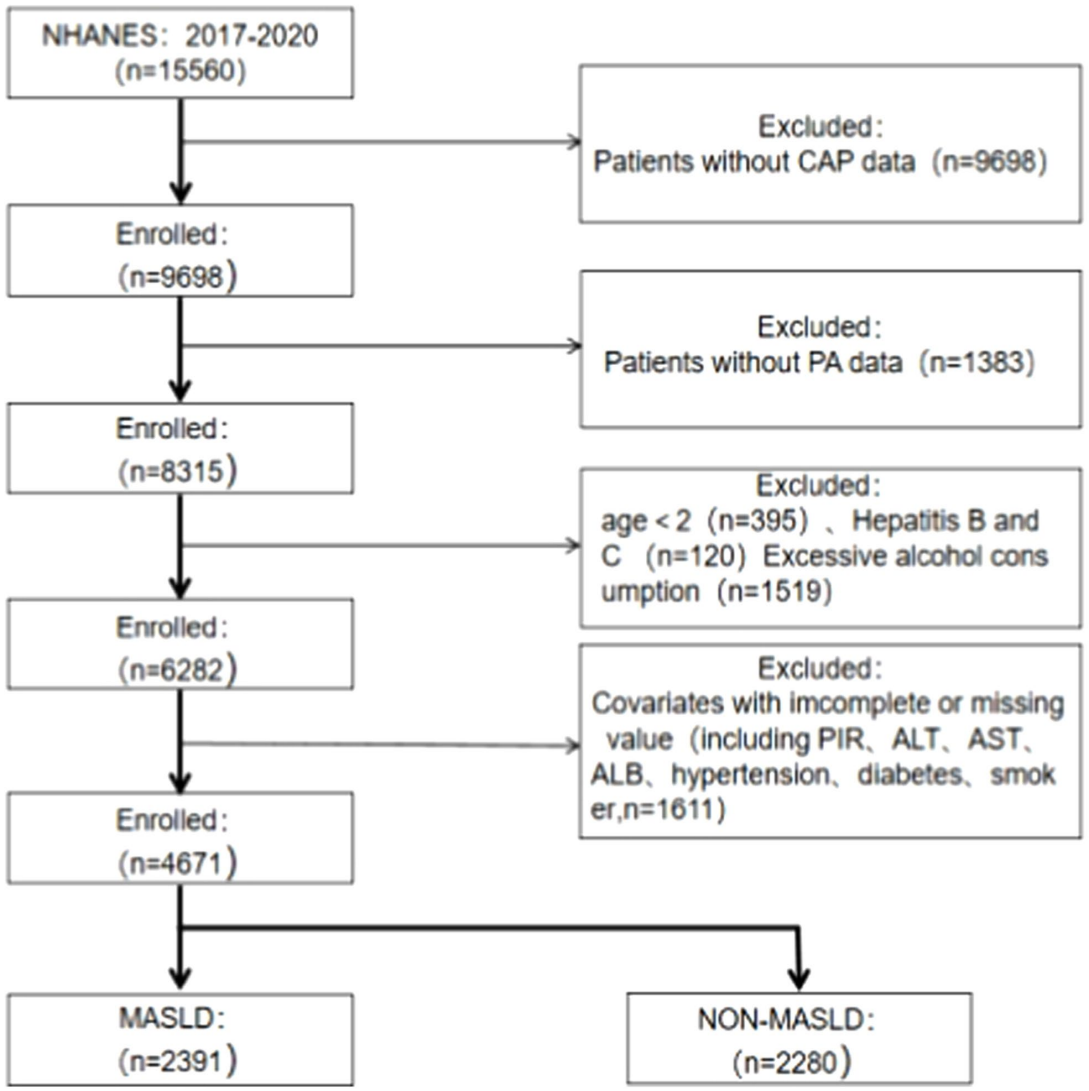
Flow diagram of the process for the selection. MASLD, metabolic dysfunction-associated steatotic liver disease; CAP, controlled attenuation parameter; PA, physical activity; PIR, poverty-to-income ratio; ALT, alanine transaminase; AST, aspartate aminotransferase; ALB, serum albumin.

Data for this study were obtained from participants in one cycle (2017–2020) of the NHANES database because vibration-controlled transient elastography (VCET) data were available in that population cycle. Liver biopsy is now the gold standard for assessing the extent of hepatic steatosis and diagnosing NAFLD. However, it has several disadvantages, including high cost, poor reproducibility, risk of bleeding, potential for infection, and even death (1:10,000) (22, 23). As a non-invasive method of assessing the prevalence and severity of NAFLD, physicians frequently use VCTE in their clinical practice. The use of controlled attenuation parameters (CAP) is very accurate in detecting hepatic steatosis and fibrosis (24). A total of 15,560 participants’ information was included in this cycle. All patients with missing physical activity questionnaire data and missing VECT results were excluded, as well as participants aged below 20 years and patients with missing covariates, as shown in Figure 1.

### 2.3 Exposure variable: PA

The frequency (number of sessions per week) and duration (length of each session) of the activities from the PA questionnaire were used to calculate the total PA. In a typical week, participants were asked how often and for how long they engaged in fitness and leisure activities lasting at least 10 min continuously, as well as strenuous and moderate physical activity. Previous studies and guidelines from health authorities suggest that 1 min of vigorous activity (VPA) is equivalent to 2 min of moderate physical activity (MPA) (12). Therefore, total PA was calculated using the following formula: Total PA = 2 × Vigorous PA + Moderate PA. Based on self-reported leisure-time PA in the relevant articles, activity patterns were categorized into four groups: (1) inactive group (did not engage in vigorous or moderate PA), (2) underactive group (reported less than 150 min of total PA per week), (3) weekend warrior group (reported at least 150 min of total PA per week with an exercise frequency of 1–2 times), and (4) the regularly active group (reported at least 150 min of total PA per week with an exercise frequency of ≥3 times) (15).

### 2.4 Outcome variable: the MASLD

Fibroscan was used to determine the degree of hepatic steatosis (25) and hepatic steatosis was defined as a controlled attenuation parameter (CAP) ≥ 264 dB/m (26), and MASLD was defined as the presence of a CAP meeting the above criteria, excluding excessive alcohol consumption (≥2 alcoholic drinks per week) and viral hepatitis (hepatitis B and C) and meet one of the following cardiometabolic risk factors for hepatic steatosis: (1) body mass index (BMI) ≥25 kg/m^2^ (23 kg/m^2^ in Asia) or waist circumference (WC) ≥94/80 cm (male/female); (2) fasting glucose ≥100 mg/dL or glycosylated hemoglobin≥5.7%, or type-2 diabetes mellitus, or being receiving treatment for type-2 diabetes; (3) blood pressure ≥ 130/85 mm Hg or on antihypertensive therapy; (4) plasma triglycerides ≥150 mg/dL or on lipid-lowering therapy; and (5) plasma high-density lipoprotein cholesterol (HDL-C) <40 mg/dL in men or < 50 mg/dL in women, or on lipid-lowering therapy (27).

### 2.5 Covariates

This study included several important covariates, referring to previous relevant NHANES cross-sectional studies (28–30), Demographic covariates included, age, sex, ethnicity (Mexican American, other Hispanic, non-Hispanic white, non-Hispanic black, or other ethnicity), education level (middle school or below, high school, college or over), and poverty-to-income ratio (PIR) (<1.3, 1.3–3.49, and ≥ 3.5). Because the diagnostic criteria for MASLD include data on BMI, waist circumference, triglycerides (TG), HDL-C, fasting blood glucose, glycated hemoglobin, and excessive alcohol consumption, to prevent data covariance in the statistical analysis, the above indicators were not included as covariates in this study, and other data, including alanine transaminase (ALT), aspartate aminotransferase (AST), and serum albumin (ALB), whether or not they were hypertensive, whether or not they were diabetic, and whether or not they were smokers were chosen. Other data, including ALT, AST, ALB, hypertension, diabetes, and smoking, were selected as covariates. Smoking status was assessed by answering the question “Have you ever smoked at least 100 cigarettes in your life?” Those who answered “yes” were defined as smokers and those who answered “no” were defined as nonsmokers. Diabetes mellitus was defined as fasting blood glucose ≥126 mg/dL or glycosylated hemoglobin ≥6.5%, self-reported physician notification of diabetes mellitus, or treatment with insulin or medication for diabetes mellitus (31). Participants with systolic blood pressure measurements of ≥130 mm Hg or diastolic blood pressure measurements of ≥80 mm Hg, self-reported physician notification of hypertension, or taking antihypertensive medication were defined as having hypertension (32).

### 2.6 Statistical methods

Baseline characteristics were described as means (95% confidence intervals) (CIs) for continuous variables and numbers (percentages) for categorical variables. Student’s *t*-test was used to compare continuous data, and chi-square test was used to compare categorical data. Logistic regression models were used to estimate the odds ratios (ORs) and 95% confidence intervals for the association between PA and MASLD. Three logistic regression models were executed to explore the association between PA and MASLD based on covariate adjustment: Model 1: Unadjusted for covariates; Model 2: adjusted for demographic variables (PIR, age, sex, ethnicity, and educational level); Model 3: Model 2 served as a base and was further adjusted for smoking status, hypertension, diabetes, and serum markers (ALT, AST, and ALB). Subgroup analyses were performed based on the following covariates: Ethnicity, sex, education level, PIR, smoking, hypertension, and diabetes. In addition, multivariate logistic regression analyses were employed to investigate the association between weekend warriors and MASLD, using participants who exercised regularly as the reference group, excluding both inactive and underactive participants. Results are presented as ORs and CIs.

All data analyses in this study were performed using EmpowerStats and R versions 4.2 and 4.1; EmpowerStats is a data analysis software designed for medical research and epidemiology, developed by the Harvard School of Public Health. It is known for its user-friendly interface and powerful statistical analysis functions and is particularly suitable for handling large-scale epidemiological data and clinical research data. All statistical manipulations were weighted according to the corresponding year-weighted data given by the NCHS analysis criteria (“WTMECPRP”); *p*-Values below 0.05 were considered statistically significant.

## 3 Results

### 3.1 Description of the study population

A total of 4,671 eligible participants were included in this study, of which 2,391 were eligible for MASLD and 2,280 for NON-MASLD, including 2,481 inactive, 695 under-active, 223-weekend warriors, and 1,262 regularly active participants. The different participants showed significant differences (*p* < 0.001) in variables, such as age; sex; education level; serum AST, ALT, and ALB levels; prevalence of hypertension; diabetes; and physical activity patterns. In this study, it was inferred from Table 1 that patients with MASLD presented an older age, a higher prevalence of males than females, a predominance of non-Hispanic white people, a higher prevalence of Americans in Mexico, higher serum ALT and AST levels, and prevalence of hypertension and diabetes, and a lower effective physical activity.

**Table 1:** Clinical characteristics of the study participants.

Variables	Total (*n* = 4,671)	NON-MASLD (*n* = 2,280)	MASLD (*n* = 2,391)	*p*-value
Age, years, mean (95% CI)	48.54(47.20, 49.88)	45.75(44.01, 47.48)	51.28(49.99, 52.56)	**<0.001**
Sex, % (95% CI)				**<0.001**
Male	45.10(43.52, 46.69)	40.40(37.51, 43.35)	49.70(47.19, 52.21)	
Female	54.90(53.31, 56.48)	59.60(56.65, 62.49)	50.30(47.79, 52.81)	
Ethnicity, % (95% CI)				**<0.001**
Mexican American	8.43(6.16, 11.43)	5.96(4.21, 8.37)	10.85(7.85, 14.81)	
Other Hispanic	7.55(5.95, 9.52)	8.27(6.43, 10.57)	6.84(5.21, 8.94)	
Non-Hispanic White	63.42(57.76, 68.73)	62.95(57.02, 68.51)	63.88(57.66, 69.66)	
Non-Hispanic Black	10.43(7.74, 13.91)	12.31(9.15, 16.36)	8.59(6.27, 11.66)	
Other ethnicity	10.17(8.13, 12.67)	10.51(8.26, 13.30)	9.84(7.70, 12.50)	
Educational level, % (95% CI)				**0.006**
Middle school or below	10.74(9.66, 11.91)	10.25(8.94, 11.73)	11.21(9.87, 12.71)	
High school	28.26(25.06, 31.69)	26.13(22.40, 30.23)	30.34(26.99, 33.92)	
College or over	61.01(57.46, 64.44)	63.62(59.01, 67.99)	58.45(54.90, 61.90)	
PIR, % (95% CI)				0.190
<1.3	20.14(18.26, 22.15)	20.27(17.70, 23.12)	20.00(18.25, 21.88)	
1.3–3.49	36.73(33.96, 39.58)	34.91(31.76, 38.18)	38.51(34.51, 42.67)	
≥3.5	43.14(39.56, 46.79)	44.82(40.28, 49.45)	41.49(37.17, 45.94)	
ALT, U/L, mean (95% CI)	21.80(21.23, 22.37)	18.25(17.67, 18.84)	25.27(24.43, 26.11)	**<0.001**
ALB, g/dl, mean (95% CI)	4.09(4.07, 4.12)	4.13(4.10, 4.16)	4.06(4.03, 4.08)	**<0.001**
AST, U/L, mean (95% CI)	20.93(20.55, 21.30)	19.97(19.52, 20.41)	21.87(21.36, 22.37)	**<0.001**
Hypertension, % (95% CI)				**<0.001**
No	62.92(59.29, 66.41)	76.16(72.23, 79.70)	49.95(46.15, 53.74)	
Yes	37.08(33.59, 40.71)	23.84(20.30, 27.77)	50.05(46.26, 53.85)	
Diabetes, % (95% CI)				**<0.001**
NO	47.38(44.83, 49.95)	62.76(59.27, 66.13)	32.33(28.87, 35.99)	
YES	52.62(50.05, 55.17)	37.24(33.87, 40.73)	67.67(64.01, 71.13)	
Smoker, % (95% CI)				0.130
Yes	38.30(35.32, 41.37)	36.13(31.88, 40.61)	40.41(36.52, 44.43)	
No	61.70(58.63, 64.68)	63.87(59.39, 68.12)	59.59(55.57, 63.48)	
PA pattern, % (95% CI)				**<0.001**
Inactive	46.94(44.36, 49.53)	39.49(36.07, 43.02)	54.22(51.41, 57.02)	
Insufficiently active	15.54(13.80, 17.46)	14.56(12.11, 17.40)	16.51(14.41, 18.84)	
Weekend warrior	6.39(4.68, 8.66)	7.50(5.19, 10.71)	5.30(3.71, 7.53)	
Regularly active	31.13(28.68, 33.70)	38.46(34.73, 42.33)	23.97(21.53, 26.58)	

For continuous variables: survey-weighted mean (95% CI), *p*-value was by survey-weighted linear regression (svyglm). For categorical variables: survey-weighted percentage (95% CI), *p*-value was by survey-weighted Chi-square test (svytable). MASLD, metabolic dysfunction-associated steatotic liver disease; PIR, poverty-to-income ratio; BMI, body mass index; ALT, alanine transaminase; ALB, serum albumin; AST, aspartate aminotransferase; PA, physical activity; OR, odds ratio; CI, confidence interval. Boldface values represent statistically significant results (*p* < 0.05).

### 3.2 Comparison between MASLD component characteristics and different PA patterns

Based on the population listed in Figure 1, we further screened the data, removing the variables with missing diagnostic indicators related to MASLD (*n*: 197), a total of 4,474 participants remained because the fasting blood glucose indicator had missing data exceeding 40%. Consequently, we did not use the interpolation method and instead chose the glycated hemoglobin indicator to observe differences in PA patterns and MASLD-related diagnostic indicators (Table 2). The results indicated differences among various PA patterns and all MASLD diagnostic indicators, and importantly, these differences were statistically significant (*p* < 0.001).

**Table 2:** Association between different PA patterns and MASDL.

Variables	Inactive	Insufficiently active	Weekend warrior	Regularly active	*p*-value
BMI, % (95% CI)					**<0.001**
<25	22.030 (18.958, 25.444)	23.985 (18.701, 30.208)	22.644 (14.907, 32.847)	31.965 (28.614, 35.514)	
≥25, <30	26.056 (23.197, 29.135)	28.325 (21.917, 35.747)	38.698 (29.698, 48.542)	33.283 (29.853, 36.901)	
≥30	51.913 (48.948, 54.865)	47.690 (40.768, 54.702)	38.658 (30.681, 47.294)	34.751 (31.459, 38.196)	
WC, mean (95% CI)	104.801 (103.604, 105.997)	102.108 (99.890, 104.326)	99.704 (97.014, 102.395)	96.432 (95.156, 97.708)	**<0.001**
Average systolic blood pressure, mean (95% CI)	122.999 (121.709, 124.288)	120.829 (119.044, 122.614)	119.702 (116.615, 122.789)	117.962 (116.556, 119.368)	**<0.001**
Average diastole blood pressure, mean (95% CI)	74.219 (73.580, 74.858)	74.912 (73.516, 76.308)	73.244 (70.674, 75.813)	71.637 (70.739, 72.534)	**<0.001**
Hypertension, % (95% CI)					**<0.001**
No	56.767 (52.119, 61.299)	63.072 (56.844, 68.892)	70.802 (59.340, 80.116)	75.073 (71.531, 78.307)	
Yes	43.233 (38.701, 47.881)	36.928 (31.108, 43.156)	29.198 (19.884, 40.660)	24.927 (21.693, 28.469)	
HbA1c, mean (95% CI)	5.860 (5.820, 5.901)	5.761 (5.612, 5.909)	5.663 (5.526, 5.800)	5.550 (5.492, 5.608)	**<0.001**
Diabetes, %(95% CI)					**<0.001**
No	39.258 (36.381, 42.212)	48.300 (40.250, 56.439)	49.312 (41.424, 57.233)	60.086 (54.496, 65.425)	
Yes	60.742 (57.788, 63.619)	51.700 (43.561, 59.750)	50.688 (42.767, 58.576)	39.914 (34.575, 45.504)	
TG, mean (95% CI)	153.129 (145.299, 160.959)	135.040 (125.195, 144.885)	153.211 (126.992, 179.430)	125.999 (119.176, 132.822)	**<0.001**
HDL-C, mean (95% CI)	50.351 (49.554, 51.149)	52.836 (51.139, 54.534)	48.446 (45.352, 51.540)	54.318 (53.023, 55.613)	**<0.001**
CAP, mean (95% CI)	276.352 (272.149, 280.555)	270.663 (262.778, 278.547)	258.351 (250.083, 266.618)	249.909 (243.787, 256.030)	**<0.001**

For continuous variables: survey-weighted mean (95% CI), *p*-value was by survey-weighted linear regression (svyglm). For categorical variables: survey-weighted percentage (95% CI), *p*-value was by survey-weighted Chi-square test (svytable). BMI, body mass index; WC, waist circumference; HbA1c, serum glycated hemoglobin; TG, triglycerides; HDL-C, high-density lipoprotein cholesterol; CAP, controlled attenuation parameter. Boldface values represent statistically significant results (*p* < 0.05).

### 3.3 Association between PA pattern and metabolic dysfunction-associated steatotic liver disease

Multivariate logistic regression analyses were conducted to explore the association between MASLD and various types of PA (as listed in Table 3). Using the inactive population as a reference, the unadjusted model (Model 1) showed that the prevalence of MASLD was lower in those who used weekend warrior (OR: 0.515, 95% CI: 0.637–1.070, *p*: 0.002) and those who used a regular exercise pattern (OR: 0.454, 95% CI: 0.377–0.546, *p* < 0.001), while the risk of MASLD in the under-exercised population was slightly but not significantly lower (*p*: 0.162). After adjusting for covariates related to age, sex, ethnicity, education level, PIR, ALT, AST, ALB, smoking, and other relevant covariates. Model 2 and Model 3 presented results that were consistent with the above findings, and in the final adjusted model, participants adopting the weekend warrior exercise pattern (OR: 0.511, 95% CI: 0.373–0.701, *p*: 0.00.6) prevalence of MASLD risk was reduced by 48.9%, participants in the regular activity population (OR: 0.621, 95% CI: 0.512–0.754, *p*: 0.00.3) prevalence of MASLD risk was reduced by 37.9%, whereas the prevalence risk in the under-exercised population was reduced by only 1.6%, which was not statistically significant (*p*: 0.896).

**Table 3:** Logistic regression modeling of different PA patterns with MASDL.

PA pattern	Model 1 OR (95% CI), *p-*value		Model 2 OR (95% CI), *p-*value		Model 3 OR (95% CI), *p-*value	
Inactive	Ref.		Ref.		Ref.	
Insufficiently active	0.826 (0.637, 1.070)	0.162	0.884 (0.691, 1.131)	0.346	0.984 (0.786, 1.234)	0.896
Weekend warrior	0.515 (0.359, 0.738)	0.002	0.497 (0.361, 0.685)	0.001	0.511 (0.373, 0.701)	**0.006**
Regularly active	0.454 (0.377, 0.546)	<0.001	0.480 (0.390, 0.590)	<0.001	0.621 (0.512, 0.754)	**0.003**

PA, physical activity; PIR, poverty-to-income ratio; BMI, body mass index; ALT, alanine transaminase; ALB, serum albumin; AST, aspartate aminotransferase; Ref. reference (1.000); OR, odds ratio; CI, confidence interval. Model 1: No covariate was adjusted; Model 2: Adjusted for age, ethnicity, education level, sex, and PIR; Model 3: Adjusted for age, ethnicity, education level, sex, PIR, diabetes, hypertension, smoker, ALT, ALB, and AST. Boldface values represent statistically significant results (*p* < 0.05).

Using the above methodology, we also examined the effect of different PA modalities on continuous type variables in the diagnostic indicators of MASLD to observe the correlation between WWs and specific metabolic indicators as well as CAP values (Supplementary Table S1) and the results showed that WWs were able to improve the CAP indicators (*β*: 258.35, 95% CI: 250.08–266.62, *p*: 0.004) and waist circumference (β: 99.70, 95% CI: 97.01–102.39, *p*: 0.014), but did not achieve the same benefits as RA in HDL-C, TG, and HbA1c.

### 3.4 Subgroup analysis

**Figure 2:**
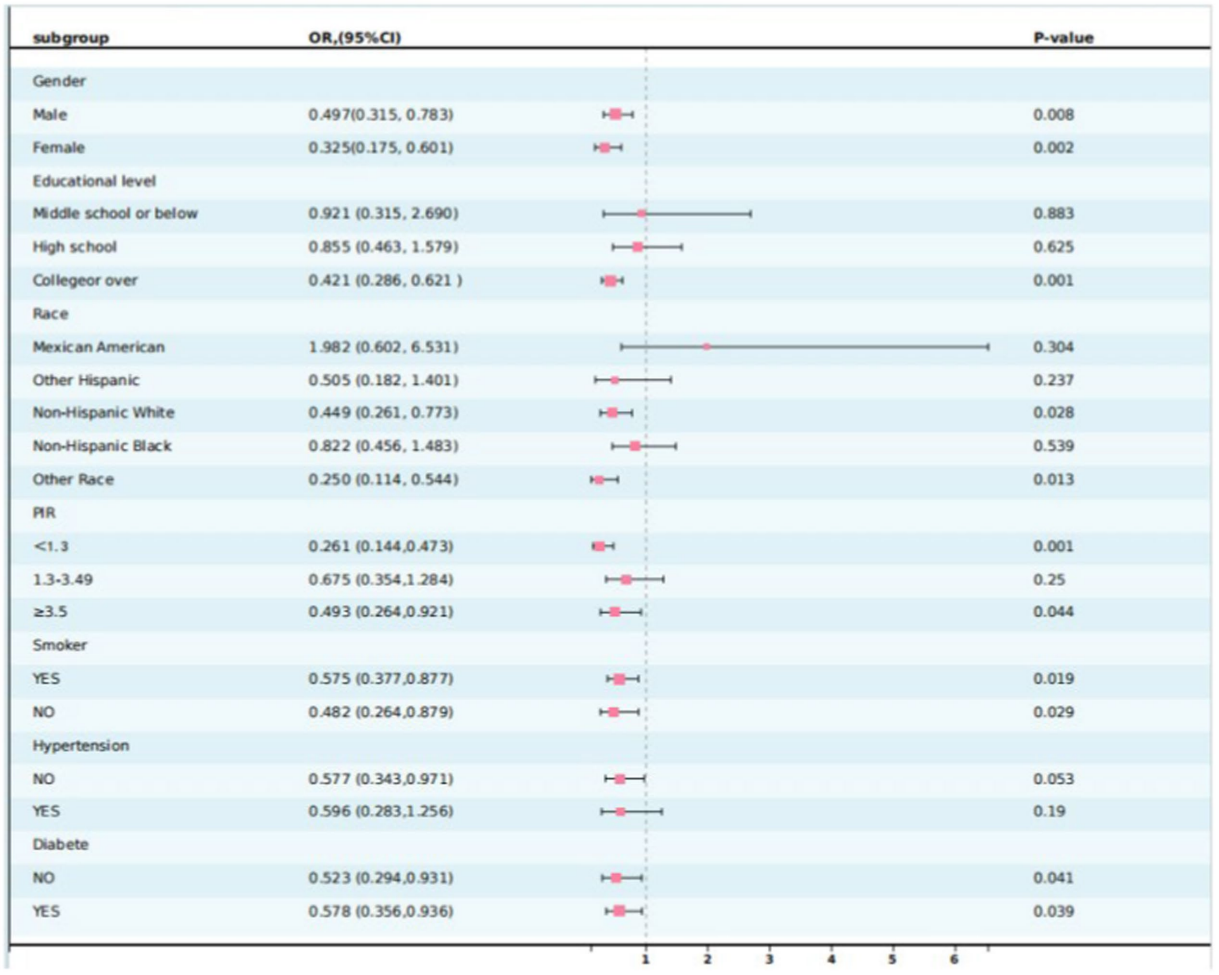
Weekend warrior subgroup analysis forest map.

To further confirm the stability of the association between PA patterns and MASLD, subgroup analyses based on the grouping of important covariates, including ethnicity, sex, education level, PIR, hypertension, and diabetes, were conducted. As shown in Table 4 and Figure 2, the association between PA patterns and MASLD remained consistent across subgroups stratified by the variables of sex, smoking, hypertension, and diabetes. Based on the results of the interaction test showing that the association between PA mode and MASLD was influenced by the level of education level, ethnicity, and PIR variables, participants who adopted the weekend warrior exercise mode had a lower risk of developing MASLD in the three subgroups mentioned above, including high levels of education (OR: 0.421, 95% CI: 0.286–0.621, *p*: 0.0001), non-Hispanic white people (OR: 0.449, 95% CI: 0.261–0.773, *p*: 0.028), and higher/lower economic income groups (OR: 0.493, 95% CI, 0.264–0.921, *p*: 0.044)/(OR: 0.261, 95% CI: 0.144–0.473, *p*: 0.001).

**Table 4:** Subgroup analysis of different PA patterns and MASLD.

Variables	Inactive (OR)	Insufficiently active (OR, 95% CI, *p*-value)	Weekend warrior (OR, 95% CI, *p*-value)	Regularly active (OR, 95% CI, *p*-value)	*p* for interaction
Sex					0.387
Male	Ref.	0.789 (0.556, 1.120) 0.201	0.497 (0.315, 0.783) 0.008	0.456 (0.368, 0.563) <0.001	
Female	Ref.	0.872 (0.628, 1.210) 0.423	0.325 (0.175, 0.601) 0.002	0.429 (0.316, 0.583) <0.001	
Educational level					**0.038**
Middle school or below	Ref.	0.778 (0.419, 1.445) 0.440	0.921 (0.315, 2.690) 0.883	0.701 (0.473, 1.039) 0.098	
High school	Ref.	0.896 (0.496, 1.620) 0.723	0.855 (0.463, 1.579) 0.625	0.508 (0.357, 0.723) 0.002	
College or over	Ref.	0.786 (0.564, 1.095) 0.177	0.421 (0.286, 0.621) 0.001	0.411 (0.320, 0.529) <0.001	
Ethnicity					**0.002**
Mexican American	Ref.	0.872 (0.525, 1.449) 0.616	1.982 (0.602, 6.531) 0.304	0.602 (0.344, 1.055) 0.127	
Other Hispanic	Ref.	2.171 (1.085, 4.340) 0.071	0.505 (0.182, 1.401) 0.237	0.451 (0.306, 0.664) 0.007	
Non-Hispanic White	Ref.	0.730 (0.501, 1.066) 0.155	0.449 (0.261, 0.773) 0.028	0.394 (0.299, 0.518) 0.001	
Non-Hispanic Black	Ref.	0.893 (0.686, 1.164) 0.436	0.822 (0.456, 1.483) 0.539	0.588 (0.433, 0.799) 0.015	
Other ethnicity	Ref.	0.825 (0.557, 1.221) 0.373	0.250 (0.114, 0.544) 0.013	0.535 (0.351, 0.818) 0.028	
PIR					**0.007**
<1.3	Ref.	0.917 (0.658, 1.280) 0.619	0.261 (0.144, 0.473) 0.001	0.409 (0.285, 0.587) <0.001	
1.3–3.49	Ref.	0.851 (0.552, 1.313) 0.478	0.675 (0.354, 1.284) 0.250	0.551 (0.439, 0.692) <0.001	
≥3.5	Ref.	0.756 (0.505, 1.131) 0.195	0.493 (0.264, 0.921) 0.044	0.396 (0.281, 0.557) <0.001	
Smoker					0.729
Yes	Ref.	0.960 (0.654, 1.409) 0.836	0.575 (0.377, 0.877) 0.019	0.525 (0.356, 0.776) 0.005	
No	Ref.	0.774 (0.566, 1.059) 0.127	0.482 (0.264, 0.879) 0.029	0.422 (0.320, 0.557) <0.001	
Hypertension					0.744
No	Ref.	0.891 (0.644, 1.234) 0.498	0.577 (0.343, 0.971) 0.053	0.501 (0.388, 0.647) <0.001	
Yes	Ref.	0.849 (0.608, 1.187) 0.352	0.596 (0.283, 1.256) 0.190	0.625 (0.470, 0.833) 0.005	
Diabetes					0.087
No	Ref.	0.949 (0.588, 1.531) 0.832	0.523 (0.294, 0.931) 0.041	0.470 (0.375, 0.590) <0.001	
Yes	Ref.	0.856 (0.581, 1.261) 0.442	0.578 (0.356, 0.936) 0.039	0.647 (0.509, 0.822) 0.002	

PIR, poverty-to-income ratio; OR, odds ratio; Ref., reference (1.000). Boldface values represent statistically significant results (*p* < 0.05).

### 3.5 Association between weekend warrior and regular activity and MASLD

Table 5 presents the results of the logistic regression analysis comparing the weekend warrior and regular activity groups. After excluding the inactive and under-active groups and using RA as a reference, the risk of developing MASLD was lower in the weekend warrior group (OR: 0.857, 95% CI: 0.548–1.339, *p*: 0.516) than in the RA group. However, there was no statistical difference between them.

**Table 5:** Association between weekend warrior and regular activity and MASLD.

**Variables**	**MASLD**		
	OR	(95% CI)	*p*-value
Physical activity pattern			
Regularly active	1.000		
Weekend warrior	0.857	(0.548–1.339)	0.516

## 4 Discussion

By analyzing information from participants in the NHANES cycle from 2017 to March 2020 (before the COVID-19 outbreak). The primary goal of this study was to look into the association between various forms of exercise and the risk of MASLD, with a particular focus on the correlation between WW exercise modes and MASLD, as well as the differences between WW exercise modes compared to RA in terms of the prevalence of MASLD. The aim is to find a convenient and effective PA mode for MASLD patients that is more in line with the rhythm of modern life.

Prior research studies have investigated the association between different PA modes on NAFLD and metabolic syndrome (28, 33), but no one has specifically investigated the correlation between WWs and MASLD risk. First, our results showed that the risk of developing MASLD was significantly lower in WWs and RA populations than in the inactive and under-exercised populations (48.9 and 37.9% risk reductions, respectively, compared to the inactive population). This indicates a notable correlation between the reduced prevalence of MASLD among participants who adopted the WWs exercise pattern. Based on these results, we also compared the effects of WWs and RA on the prevalence of MASLD. Our findings showed that although the risk of MASLD was slightly lower in WW participants than in RA participants, there was no significant difference between the two (P: 0.516). This suggests that WWs, as a novel exercise modality, could provide the same benefits as RA in combating MASLD. This finding is consistent with a previous study on NAFLD, which indicated that the risk of NAFLD decreased significantly with increased regular physical activity. Specifically, under moderate or vigorous exercise patterns, the “weekend warrior” group exhibited a 45% reduction in disease risk compared to the inactive group (OR: 0.55, 95% CI: 0.44–0.67), with comparable efficacy to that of regular exercise.

The baseline level effectively reflected the characteristics of MASLD patients, with over half of the participants diagnosed with MASLD, which closely correlates with the rising prevalence of obesity, type-2 diabetes, and metabolic syndrome in the global population. Compared to the non-MASLD population, there was a higher prevalence of males than females, older participants, and lower rates of higher education. Educational level has been identified as a protective factor for NAFLD. Those who receive a high level of education tend to be more concerned about their health and more inclined to make lifestyle adjustments to prevent the disease. Studies have shown that higher levels of education are associated with a reduced risk of NAFLD. People with less education are more likely to suffer a number of health issues because they have less access to healthcare and have fewer healthy lifestyle choices (34, 35). Furthermore, educational attainment is strongly associated with economic status, and individuals with low educational income levels are more likely to face challenges with subsistence work, resulting in heightened stress and economic hardships, which consequently contributes to an increase in metabolic syndrome (36). The prevalence appears to be higher in Mexican Americans, which is consistent with previous studies (37), and it is considered that it may be associated with variant I148M of the PNPLA3 gene, which, as by far the gene most strongly associated with genetic susceptibility to NAFLD, has been shown by relevant studies to be more common in Mexican Americans (38). Therefore, in addition to lifestyle differences, the complexities of genetic inheritance also significantly affect the occurrence and development of MASLD, which, of course, is beyond the scope of this study.

Furthermore, it is essential to consider how the type of exercise affects the improvement of MASLD. A meta-analysis evaluating the duration, frequency, and intensity of physical activity interventions (including both aerobic and anaerobic exercises) demonstrated that physical activity effectively improves BMI and hepatic lipid levels in obese patients. However, the analysis did not reveal significant differences between the two types of exercises in terms of their efficacy (39). Nevertheless, it is undeniable that energy expenditure during aerobic exercise is generally higher than that during resistance training (40). Additionally, studies have shown that the “dose” and intensity of aerobic exercise do not significantly differ in their ability to reduce hepatic fat content (41). In this study, we focused solely on the total duration and frequency of physical activity without distinguishing between types of exercises. Moreover, the duration of exercise shows a ceiling effect, indicating that exceeding a certain threshold does not provide additional benefits. These factors might contribute to minor discrepancies.

Through the description of the study population comparing the diagnostic indicators of metabolic dysfunction by different physical activity patterns, we can see that all the observed indicators have significant differences (*p* < 0.01) across different exercise modes. This reflects the benefits of effective exercise modes on metabolic function indicators and supports previous findings that active individuals are less likely to suffer from metabolic syndrome (42). Furthermore, PA is effective in improving blood pressure, glycated hemoglobin, obesity, high triglycerides, LDL-C levels, and other diagnostic indicators (43); however, there are relatively few studies examining the improvement of weekend warriors on metabolic syndrome. The results slightly differ from this study, where we found that the WWs mode was not universally effective in improving diagnostic indicators of MASLD. There were significant improvements in liver fat deformation and waist circumference, but WWs did not achieve the same results as RA in indices, such as HDL-C, TG, and HbA1c (Supplementary Table S1).

The frequency and duration of PA are the primary distinctions between WWs and RAs; therefore, it is possible that individuals with WW patterns engaged in much more than 150 min of exercise. It is also important to note that some individuals who achieved the 150-min mark performed high-frequency physical activity throughout the week. Relevant studies have demonstrated that high-frequency counts of physical activity are more effective in improving HbA1c levels, whereas high-intensity physical activity did not reduce HbA1c levels (44). Furthermore, the increase in PA and HDL-C levels occurred gradually, and the association was only statistically significant when individuals engaged in physical activity 3–5 times per week and consumed between 1,200 and 1,600 kcal of energy (45), suggesting that engaging in physical activity only 1–2 times per week (weekend warriors) may not effectively improve metabolic markers on its own. A cross-sectional study from KNHANES suggested that WWs did not reduce the incidence of metabolic syndrome (28). However, another survey of weekend warriors among Chinese farming adults presented different results, indicating that WWs can prevent metabolic syndrome; reduce triglycerides, blood pressure, and fasting blood glucose levels; and improve waist circumference (43). Part of the reason for this discrepancy is attributed to differences in the study populations; the primary study population described in this article is the European population, while the above case study population is the Asian population. Nevertheless, it is undeniable that there are limitations to the effect of WWs on individual metabolic indices, and further analysis may require a broader range of countries and regions as well as a larger base of participants.

The results of subgroup analyses showed that the associations between different modes of PA and MASLD were consistent across majority of the groups, whereas educational level, PIR, and ethnicity variables significantly modified the associations, and cardiometabolic risk was strongly correlated with educational attainment, with lower levels of educational attainment being associated with higher cardiometabolic risk (46). The benefits of RA on MASLD are good in middle and higher-education populations, but the PA pattern of WWs shows differences only in higher-education populations. A study from Finland showed that physical activity level was positively correlated with the return on education in adulthood (47), Therefore, we hypothesized that populations with higher levels of education may have a better understanding of exercise during adolescence and that the weekend warrior physical activity model may be more widely adopted and accepted among these populations, who are better equipped to recognize and use the weekend warrior exercise strategy.

The key strength of this study is that it is the first to investigate the association and differences among various exercise modalities on MASLD, particularly to in providing a reference regarding whether WWs can be a new option for improving MASLD. Second, we selected data from 2017 to 2020, and unlike other years, Fibroscan was used to assess hepatic steatosis to achieve a better diagnosis of MASLD, which is still not as accurate as liver biopsy, but it is one of the more authoritative diagnostic tools available. The findings of this study may offer additional options and preventive strategies for MASLD patients and clinicians.

Although the statistical approach and inclusion and exclusion criteria were strictly followed during this study, it is inevitable that there remains significant room for improvement. First, as a large-scale population-based cross-sectional study from the NHANES database, only associations between the exposure variables and the outcome variables can be observed, causality between the two cannot be directly inferred, and further detailed studies are needed to verify these associations. Second, our exposure indicators were obtained in the form of questionnaires, and participants inevitably experienced recall bias, which may have introduced potential confounders and bias to our study. The distribution of our participants did not involve many cycles to select more accurate measures of outcome indicators. Furthermore, we excluded participants under 20 years of age, resulting in a population base that was small and did not provide a relevant reference value for adolescents with MASLD. Finally, although we excluded confounders as much as possible and included relevant covariates to refine our study, it was not possible for us to completely eliminate unmeasured confounders. Since the diagnosis of MASLD involves several indicators, we chose not to include more covariates to prevent biased results due to covariance between variables. We need more accurate measurement and selection of variables, along with in-depth studies to understand the contribution of WWs to MASLD and the exploration of related mechanisms.

## 5 Conclusion

In summary, our results show that WWs’ activity patterns and their risk of MASLD are significantly correlated and that they can improve MASLD with benefits comparable to those of RA. This provides additional options for individuals with MASLD who are unable to meet the recommended criteria in the exercise guidelines, as well as treatment options for clinicians.

## Data Availability

The datasets presented in this study can be found in online repositories. The names of the repository/repositories and accession number(s) can be found at: https://www.cdc.gov/nchs/nhanes/index.htm.
