# Supplementary material for: Association between weekend warriors and MASLD—a cross-sectional study of the NHANES database 2017–2020
Source: Front Med (Lausanne). 2025 Apr 2;12:1531437. doi: 10.3389/fmed.2025.1531437 (PMC11999941; doi:10.3389/fmed.2025.1531437)
Supplement: Supplementary file 1 [file Table_1.DOCX]

Supplementary Table 1

| **Variables** | **Inactive**  **β（95%CI）** | **Insufficiently active**  **β (95%CI) *P*-value** | **Weekend warrior**  **β (95%CI) *P*-value** | **Regularly active**  **β (95%CI) *P*-value** |
| --- | --- | --- | --- | --- |
| **CAP** | 276.35  (272.15,280.55) | 270.66  (262.78,278.55)  0.896 | 258.35  (250.08,266.62)  **0.004** | 249.91  (243.79,256.03)  **0.004** |
| **WC** | 104.80  (103.60 ,106.00) | 102.11  (99.89 ,104.33)  0.434 | 99.70  (97.01,102.39)  **0.014** | 96.43  (95.16,97.71)  **0.002** |
| **HbA1c** | 5.86  (5.82,5.90) | 5.76  (5.61,5.91)  0.801 | 5.66  (5.53,5.80)  0.825 | 5.55  (5.49,5.61)  **0.033** |
| **TG** | 153.13 (145.30,160.96 | 135.04  (125.19,144.89)  0.104 | 153.21  (126.99,179.43)  0.975 | 126.00  (119.18,132.82)  **0.014** |
| **HDL-C** | 50.35 (49.55 ,51.15) | 52.84  (51.14,54.53)  0.340 | 48.45  (45.35,51.54)  0.753 | 54.32  (53.02,55.61)  **0.004** |

**Abbreviations：** CAP， controlled Attenuation Parameter； WC: waist circumference； HbA1c, serum glycated hemoglobin； TG: triglycerides; HDL-C: high-density lipoprotein cholesterol; CI， confidence interval.
